# Assessing Preclinical Diabetic Retinopathy: The Role of Optical Coherence Tomography Angiography

**DOI:** 10.7759/cureus.72747

**Published:** 2024-10-31

**Authors:** Pravallika Pamulapati, Manmath K Das, Gayatree Mohanty

**Affiliations:** 1 Ophthalmology, Kalinga Institute of Medical Sciences, Bhubaneswar, IND

**Keywords:** capillary dropouts, diabetic retinopathy, flow void areas, foveal avascular zone, optical coherence tomography angiography, perfused capillary density, retinal microvasculature

## Abstract

Objective

The present study aimed to investigate the variations in retinal microvascular parameters among diabetic patients without diabetic retinopathy (No DR), those with nonproliferative diabetic retinopathy (NPDR), and healthy controls using spectral domain optical coherence tomography angiography (SD-OCTA).

Methods

An observational cross-sectional case-control study was conducted involving 68 eyes classified into three groups: 32 eyes with No DR, 16 eyes with NPDR, and 20 controls. The study assessed parameters such as perfused capillary vessel density, foveal avascular zone (FAZ) area, mean vessel diameter, number of capillary dropouts, and flow void areas. OCTA images were obtained using the SD-OCT system (Spectralis HRA, Heidelberg Engineering, Germany) and analyzed with custom MATLAB software. Statistical analysis was performed using SPSS Statistics (IBM Corp., Armonk, NY), with significance set at p<0.05.

Results

Significant differences were observed across the groups for perfused capillary density, FAZ area, number of capillary dropouts, and number of flow void areas. Specifically, NPDR patients exhibited a significantly lower perfused capillary density (36.50%) and No DR patients demonstrated a considerably higher perfused capillary density (43.98%) when compared with controls (42.71%; p<0.001). The FAZ area was significantly larger in NPDR patients (0.5981 mm²) compared to the No DR (0.3581 mm²) and controls (0.3550 mm^2^; p<0.001). The number of capillary dropouts and flow void areas were significantly higher in NPDR patients but were also detected in the No DR group (p<0.001). No significant differences were found in mean vessel diameter among the groups (p=0.061).

Conclusions

The study confirms that NPDR is associated with distinct changes in retinal microvascular parameters compared to both No DR and control groups. These changes include reduced perfused capillary density, increased FAZ area, and elevated numbers of capillary dropouts and flow void areas. It also confirms that No DR can be associated with increased capillary vessel density as well as with capillary dropouts and void areas. OCTA proves to be a valuable instrument for evaluating retinal microvascular changes and could aid in the early detection and treatment of diabetic retinopathy (DR).

## Introduction

Diabetes mellitus is a chronic, progressive disorder characterized by high blood sugar levels [1]. The estimated global population of people with diabetes is expected to reach 710 million by 2046, comprising 10.8% of the total population [2]. The inadequate management of diabetes can lead to vascular damage, which can be classified into two categories: macrovascular complications that affect the body's large blood vessels, including coronary artery disease, peripheral artery disease, and stroke; and microvascular complications that impact the small blood vessels, such as retinopathy, neuropathy, and nephropathy. These complications are the primary reasons for sickness and mortality among people with diabetes [3].

Diabetic retinopathy (DR) is a major cause of vision loss globally and is the leading factor behind blindness among working-age adults in developed nations. Its impact on quality of life is substantial [4]. In 2010, DR was recognized as the fifth most common reason for preventable vision impairment worldwide, accounting for 1.9% of visual impairment and 2.6% of blindness [5]. The circulatory changes in diabetic patients are identified using several techniques that assess circulatory function and analyze different parts of the eye's tissue circulation [6-10].

Currently, there is no consensus regarding blood flow variations during different stages of DR. This study discusses the use of optical coherence tomography angiography (OCTA) as an innovative method for evaluating blood vessel perfusion in the human eye. This technique has been employed to investigate the perfusion of retinal and choroidal blood vessels in patients with diabetes [11]. The OCTA reports of diabetes patients show both qualitative and quantitative variations in blood vessel perfusion in the retina and choroid. They also demonstrate how different treatments affect ocular blood perfusion and its potential utility in early diagnosis and risk assessment of patients with DR [12].

## Materials and methods

Study design

The study was an observational cross-sectional case-control research conducted from September 2022 to April 2024 in the Department of Ophthalmology, Pradyumna Bal Memorial Hospital, KIMS, Bhubaneswar. A total of 68 eyes were examined, comprising 32 eyes from diabetic patients without diabetic retinopathy (No DR), 16 from diabetic patients with nonproliferative diabetic retinopathy (NPDR), and 20 from controls. The Institutional Ethics Committee approved the study protocol. All processes adhered to the principles of the Declaration of Helsinki, and all subjects provided informed consent before participating in the study. Patients aged 15-75 years with diabetes mellitus were included in the study. Exclusion criteria comprised individuals with essential hypertension, peripheral vascular disorders, Raynaud’s disease, systemic sclerosis, lupus, rheumatoid arthritis, mixed connective tissue disorder, dermatomyositis, polymyositis, and dyslipidemia.

Study procedure

The demographic information of all qualified subjects was documented; clinical history was taken and a comprehensive ophthalmic examination was performed, which included a visual acuity assessment, slit lamp examination, dilated fundoscopy examination with 90D lens, and indirect ophthalmoscopy. The patients were classified based on the severity of DR according to the Early Treatment Diabetic Retinopathy Study (ETDRS) and staging system. They then underwent OCTA imaging.

Optical Coherence Tomography Angiography Image Acquisition

Utilizing a commercial spectral-domain OCT system (Spectralis HRA Heidelberg Engineering, Germany), all OCTA images with a 3 x 3 mm macular area were captured [13]. This system has two light sources: an 820 nm laser and an 870 nm SLD light source. It is capable of performing 40,000 A-scans per second and provides a digital axial resolution of 3.9 μm, a transverse resolution of 14 μm, and a scanning depth of 1.9 mm. Additionally, it offers a maximum field of view of 30 x 30 degrees, 11 μm high-speed mode isotropic resolution, and 5 μm isotropic high-resolution mode. With a lateral resolution of 5.7 μm/pix, the SPECTRALIS® OCT Angiography Module produces high-quality OCTA pictures. When combined with the accuracy of TruTrack Active Eye Tracking, the OCTA Module makes it possible to see intricate capillary networks in remarkable detail. The axial resolution of 3.9 μm/pixel allows for the segmentation of the choriocapillaris layer and the histologically confirmed retinal vascular plexuses, including the superficial, intermediate, and deep capillary plexuses. Custom slabs provide a more thorough clinical assessment of the superficial and deep vascular plexuses [14].

Optical Coherence Tomography Angiography Image Analysis

**Figure 1:**
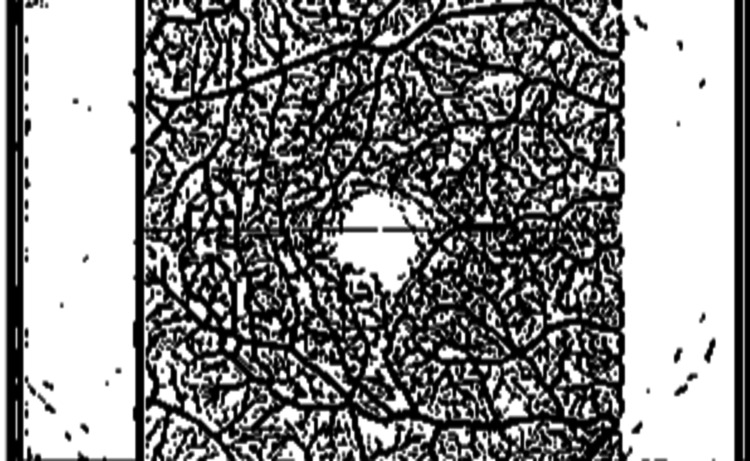
Binarized image formed after processing and segmentation of the OCTA MIP image MIP: maximum intensity projection; OCTA: optical coherence tomography angiography

**Figure 2:**
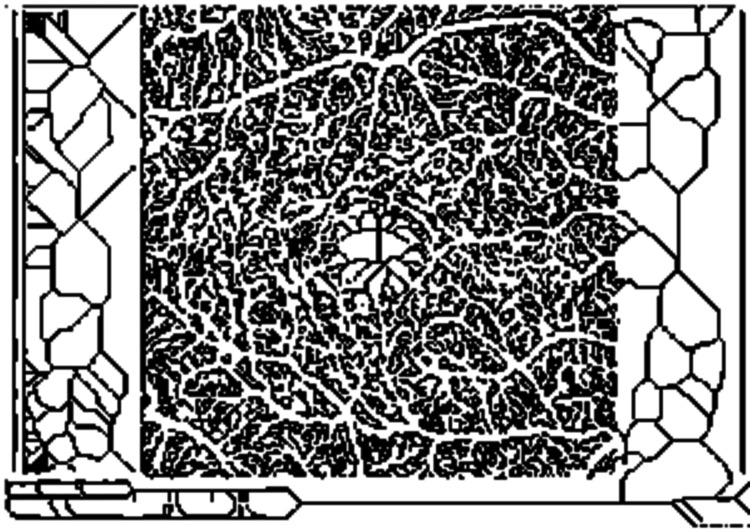
The network is skeletonized forming a skeletonized image

OCTA images were analyzed using specialized MATLAB software (The Mathworks, Inc., Natick, MA) [13]. A maximum intensity projection (MIP) image was loaded into the OCTAVA (OCTA Vascular Analyser) toolbox [15]. Images were first pre-processed in MATLAB using an optional median filter to reduce noise and a Frangi filter to enhance the intensity of vessel-like structures. Images were segmented into two regions representing “vessels” and “not vessels” using the adaptive threshold method to create a binary mask (Figure 1). This binarized image was sent to ImageJ and the network was skeletonized using a MATLAB built-in 3D thinning algorithm, and a heatmap of vessel diameter was generated using a Euclidian distance transform, and the skeletonized image was formed (Figure 2).

**Figure 3:**
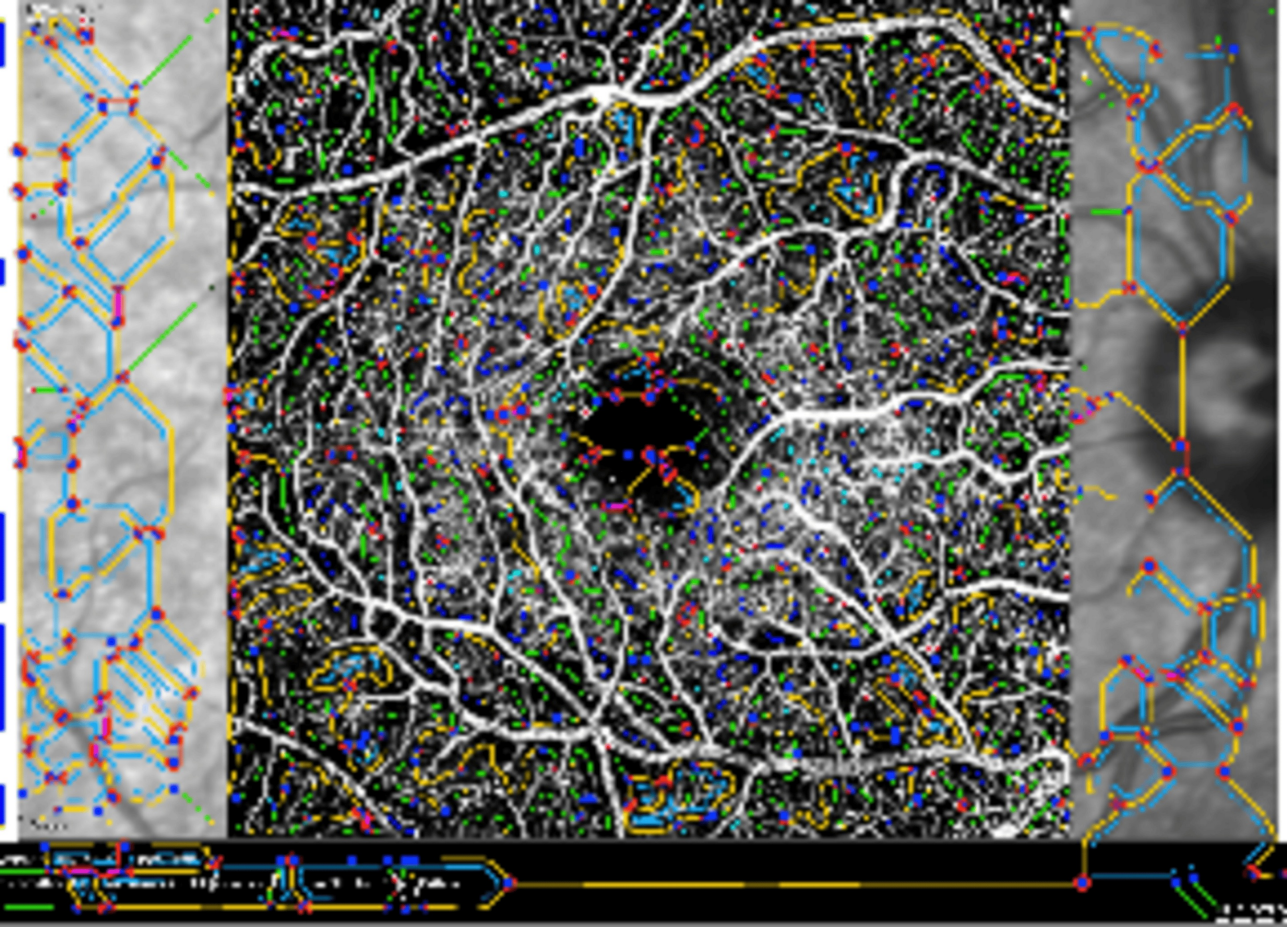
The network is displayed overlaid on the original MIP image forming an overlay image The colors represent the interconnectivity with the network MIP: maximum intensity projection

**Figure 4:**
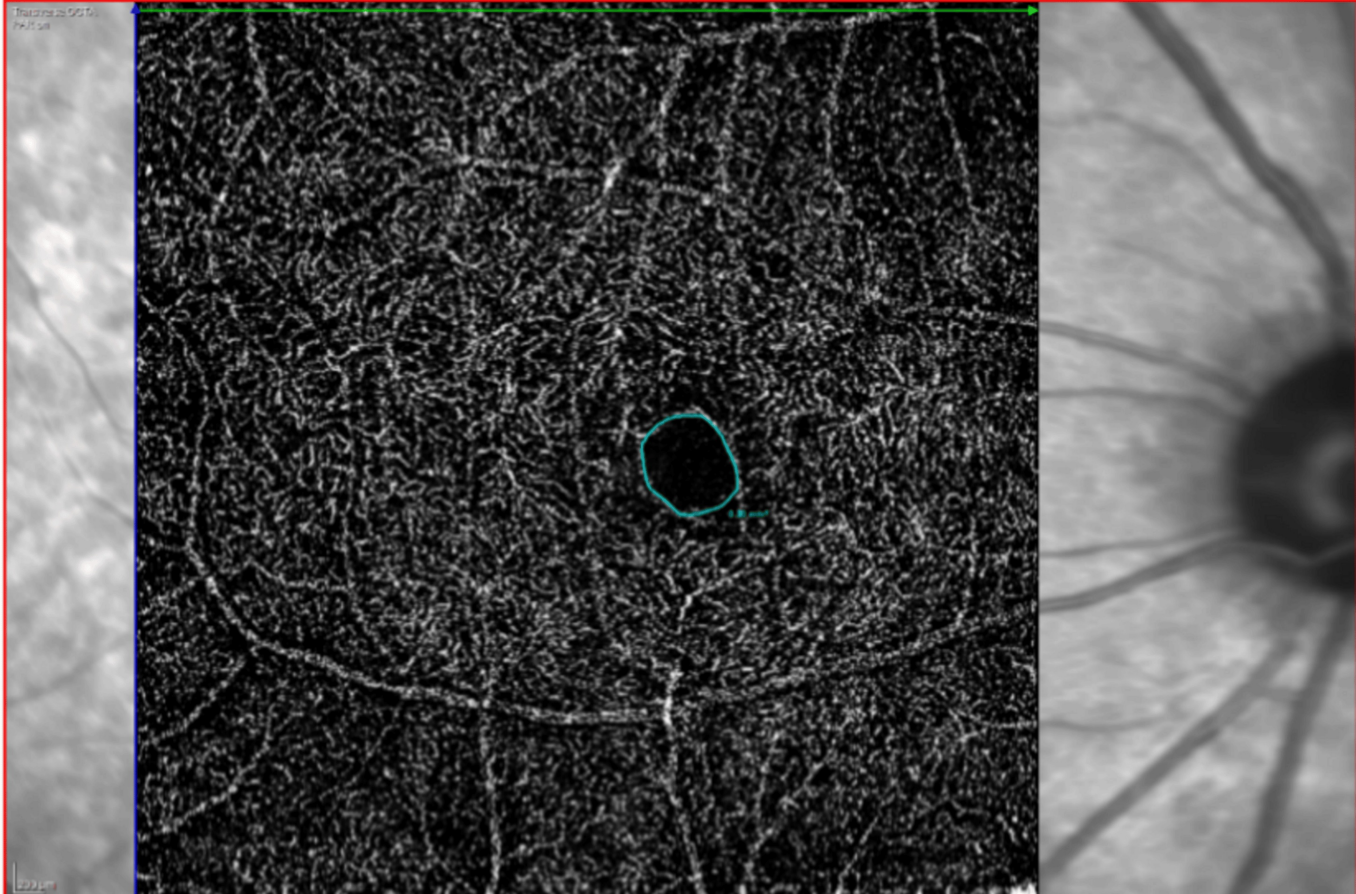
OCTA picture at the intermediate capillary plexus layer depicting the region overlay tool used to calculate the FAZ area in a No DR patient FAZ: foveal avascular zone; No DR: without diabetic retinopathy; OCTA: optical coherence tomography angiography

**Figure 5:**
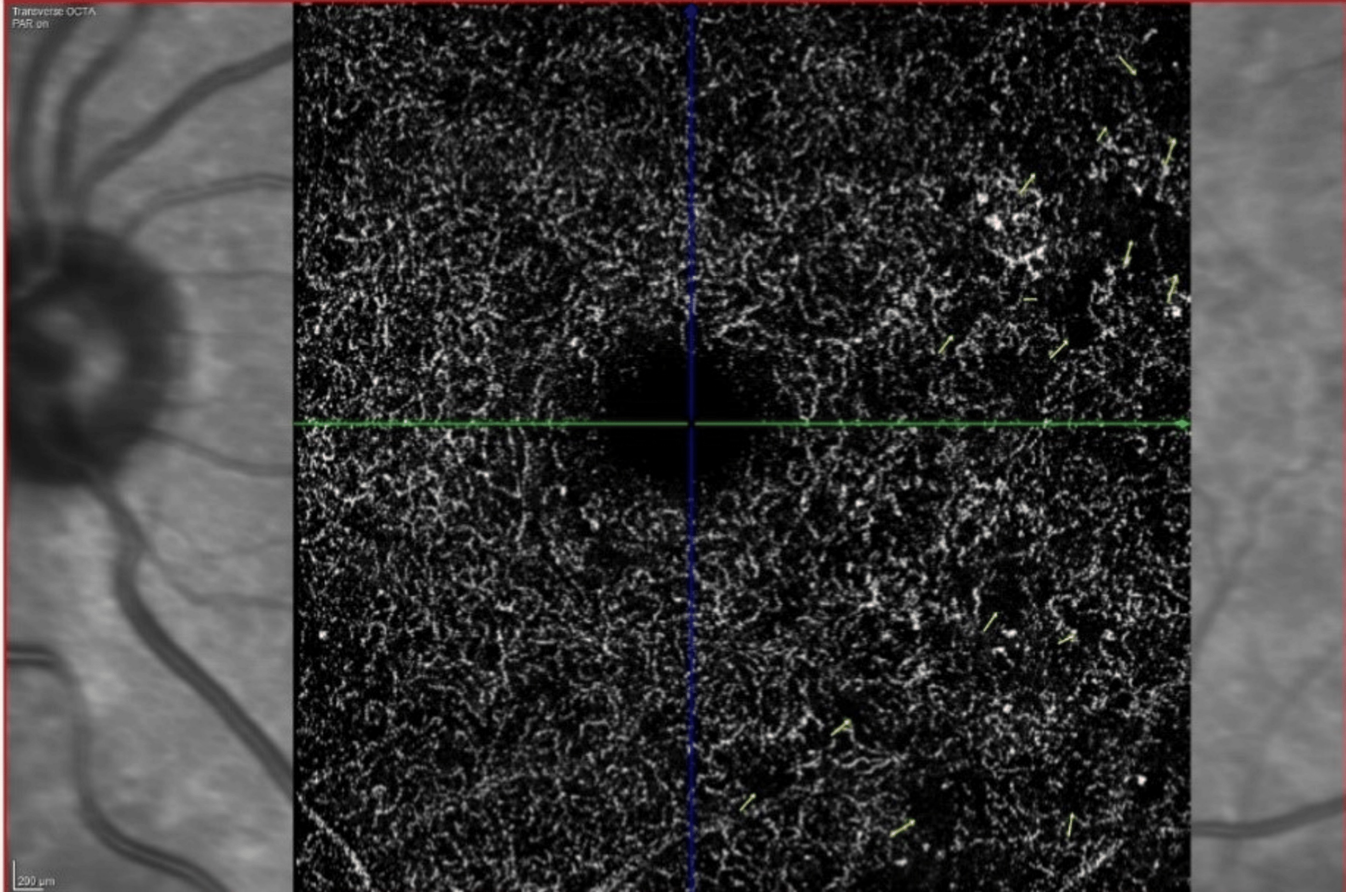
OCTA picture at the deep capillary plexus layer in an NPDR patient The yellow arrows depict the capillary dropouts NPDR: nonproliferative diabetic retinopathy; OCTA: optical coherence tomography angiography

**Figure 6:**
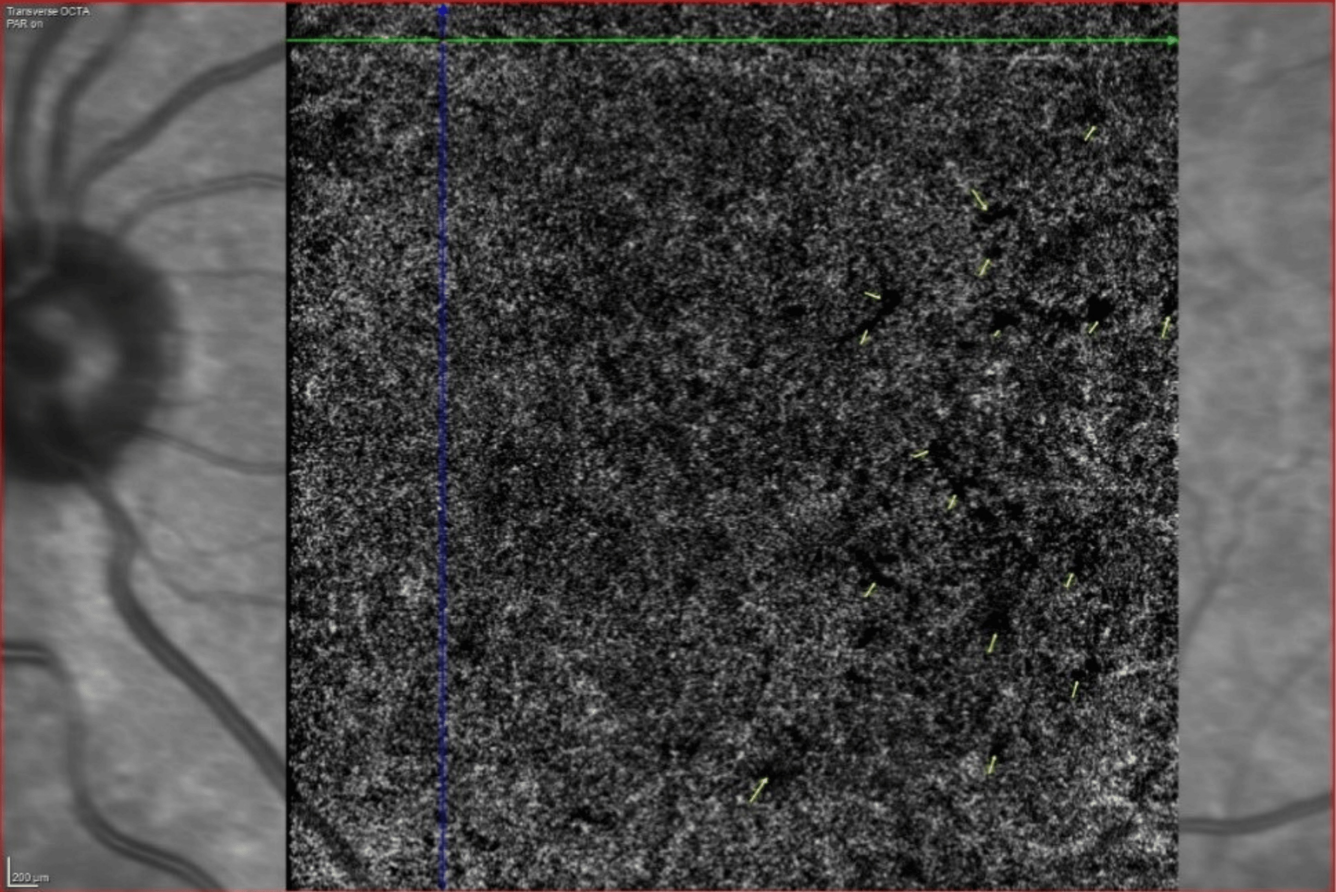
OCTA picture at the choriocapillaris layer in an NPDR patient The yellow arrows depict the flow void areas NPDR: nonproliferative diabetic retinopathy; OCTA: optical coherence tomography angiography

The nodes and endpoints were identified, and vessels were categorized by their interconnectivity to the network. The network was displayed overlaid on the original MIP image forming an overlay image (Figure 3). Finally, these outputs were then analyzed using the MATLAB software, to generate and compile metrics of the network by which perfused capillary vessel density and mean vessel diameter were acquired. The foveal avascular zone (FAZ) area was calculated at the intermediate capillary plexus layer using the region overlay tool provided built into the machine (Figure 4). The number of capillary dropouts was calculated at the deep capillary plexus layer (Figure 5). The number of flow void areas was calculated at the choriocapillaris layer (Figure 6).

Statistical analysis

The statistical analysis was performed using SPSS Statistics version 23.0 (IBM Corp., Armonk, NY). All continuous variables were expressed by mean ± standard deviation (SD) and categorical variables by frequency and percentage. For group comparison, the student t-test was used. For measuring the association between categorical variables, chi-square test statistics were used. A p-value <0.05 was considered statistically significant.

## Results

Our study included a total of 68 eyes classified into the following groups: No DR, NPDR, and controls. Table 1 presents the demographic data. The No DR group comprised 32 eyes, with 18 being male and 14 being female, and a mean age of 51.41 ± 8.012 years. The NPDR group consisted of 16 eyes, with 10 being male and six being female, and a mean age of 60.06 ± 6.618 years. The control group included 20 eyes, with an equal split of 10 male and 10 female, and a mean age of 44.30 ± 8.850 years.

**Table 1:** Demographic data No DR: without diabetic retinopathy; NPDR: nonproliferative diabetic retinopathy; SD: standard deviation

Variables	No DR	NPDR	Control
No. of eyes	32	16	20
Male/female	18/14	10/6	10/10
Age, years, mean ± SD	51.41 ± 8.012	60.06 ± 6.618	44.30 ± 8.850

Table 2 shows the distribution of parameters across the groups.

**Table 2:** Distribution of parameters across groups FAZ: foveal avascular zone; No DR: without diabetic retinopathy; NPDR: nonproliferative diabetic retinopathy

Parameter	Group	N	Mean	Std. deviation	P-value
Perfused capillary density, %	No DR	32	43.98	1.07	<0.001
	NPDR	16	36.50	1.09	
	Control	20	42.71	0.90	
FAZ area, mm^2^	No DR	32	0.3581	0.11	<0.001
	NPDR	16	0.5981	0.11	
	Control	20	0.3550	0.11	
Mean vessel diameter, microns	No DR	32	70.88	19.55	0.061
	NPDR	16	80.19	22.91	
	Control	20	65.60	9.29	
No. of capillary dropouts	No DR	32	3.75	5.430	<0.001
	NPDR	16	21.06	13.557	
	Control	20	0.00	0.000	
No. of flow void areas	No DR	32	6.66	7.110	<0.001
	NPDR	16	25.94	18.336	
	Control	20	0.00	0.000	

The density of perfused capillaries was notably lower in patients with NPDR compared to the No DR group and the control group. Conversely, in No DR patients, the density was higher compared to both the control and NPDR groups, with mean values of 43.98% ± 1.07 in No DR, 36.50% ± 1.09 in NPDR, and 42.71% ± 0.90 in the control group (p<0.001). The FAZ area was notably larger in patients with NPDR compared to the No DR group and the control group. There was no significant difference in the FAZ area between the No DR and control groups, as evidenced by mean areas of 0.3581 mm² ± 0.11 in the No DR group, 0.5981 mm² ± 0.11 in the NPDR group, and 0.3550 mm² ± 0.11 in the control group (p<0.001).

The data indicates no significant variance in vessel diameter among the No DR, NPDR, and control groups, as the difference in mean vessel diameter did not reach statistical significance (p=0.061). The number of capillary dropouts was significantly higher in NPDR (21.06 ± 13.56) patients compared to both the No DR (3.75 ± 5.43) group and the control (0.00 ± 0.00) (p<0.001). The number of flow void areas was significantly higher in NPDR (25.94 ± 18.34) patients compared to both the No DR (6.66 ± 7.11) group and the control (0.00 ± 0.00) group (p<0.001).

## Discussion

Regarding the group-wise distribution of mean perfused capillary density (%), the present study observed the highest value of mean cases in the No DR group (43.98%) followed by the control group (42.71%) and a decrease in the NPDR group (36.50%). Overall, the difference between the groups was statistically significant. The study by Ong et al. [16] found no significant difference between the OCTA values of diabetic eyes without retinopathy and those of healthy eyes. In contrast, Rosen et al. [13] reported increased capillary density in the full retina in eyes with No DR compared to healthy eyes. 

In terms of the group-wise distribution of mean FAZ area (mm^2^), the highest value was observed in the NPDR group (0.5981 mm^2^) followed by values that were more or less similar in the control group (0.3550 mm^2^) and the No DR group (0.3581 mm^2^). Overall, we found the difference between the groups to be statistically significant. Johannesen et al. [17] conducted a systemic review involving eight studies investigating the changes in the FAZ in DR patients. Seven of these studies found that the FAZ in NPDR patients was larger compared to the healthy control group. A similar study by Kim et al. [18] also observed that there was a positive correlation between the severity levels of DR and the FAZ area and FAZ perimeter.

As for the group-wise distribution of the number of capillary dropouts, the highest mean value was observed in the NPDR group (21.06) followed by the No DR group (3.75) and the control group (0.00) in our study. Overall, we observed that the difference between the groups was statistically significant. The study by Chua et al. [19] found comparable contextual outcomes, and the researchers confirmed that diagnosing DR eyes requires precise differentiation “of low contrast areas from abnormal capillary dropout." Previous qualitative studies by Couturier et al. [20], Ishibazawa et al. [21], and Soares et al. [22] on DR have shown that OCTA is capable of delineating retinal capillary nonperfusion with better resolution than fluorescein angiography, providing an improved visualization of capillary dropout and changes in the FAZ.

In the present study, in the group-wise distribution of flow void areas, where highest mean value was found in the NPDR group (25.94) followed by the No DR group (6.66) and the control group (0.00), and the difference was statistically significant. The study by Coscas et al. [23] in this particular dimension noticed that the diabetes patient had many flow void areas that are readily apparent, but the healthy eye possesses essentially none of these flow void areas. In terms of the group-wise distribution of mean vessel diameter (µm), the highest value was observed in the NPDR group (80.19 µm) followed by the No DR group (70.88 µm) and the Control group (65.60 µm). The difference between the groups was not statistically significant. Dimitrova et al. [24] and Tey et al. [25] also showed vessel density decreases in patients with DR, as well as a diabetic patient without DR, which aligns with our findings.

Limitations of the study

A major drawback of this study was its limited duration, due to which a larger sample size could not be obtained. This was a hospital-based study and thus may not include patients seen in the community; hence, the findings may not be generalized. The control group was younger than the diabetic age group, which could lead to biased comparisons and limit the validity of the conclusions drawn about the effects of diabetes on the variables studied. The number of patients with isolated diabetes mellitus was low. They mostly had comorbidities like hypertension and hyperlipidemia. This can complicate interpretations, as it may be difficult to isolate the effects of diabetes alone, further hindering the ability to make targeted recommendations for managing diabetes without other complications. Motion artifacts made grading in certain patients impossible as this can lead to misclassification or inaccuracies in the outcomes, ultimately affecting the study's findings. Further clinical correlation could not be made due to the short follow-up period as, without long-term data, it is difficult to determine how the observed parameters - like perfused capillary density or FAZ area - change as the disease progresses or how treatment impacts these metrics over time.

## Conclusions

Our findings highlight the significance of OCTA in assessing retinal vascular changes in diabetic patients, demonstrating its efficacy in identifying key metrics such as perfused capillary density, FAZ area, capillary dropouts, and flow void areas. Traditionally, DR has been classified using the ETDRS severity scale, based on photographic assessments and standard color fundus images. However, our study highlights the detection of these microvascular changes before the appearance of fundus changes in DR (preclinical DR). Diagnosis of such preclinical DR changes is clinically relevant for two reasons: it guides the physician to implement more intensive control of blood sugar levels, and it helps determine whether these changes can be reversed with aggressive glycemic management.

Currently, the medical literature lacks documented evidence as to whether such preclinical DR changes can be reversed. Our findings underscore OCTA's potential for early diagnosis and monitoring of DR, providing valuable insights into the microvascular alterations associated with this condition. Integrating OCTA into clinical practice offers a promising advancement for both routine clinical use and clinical trials for a more comprehensive evaluation of retinal health in diabetic patients. Future research should focus on exploring OCTA’s capabilities further, particularly in guiding treatment strategies and screening guidelines. While the ETDRS and International Clinical Diabetic Retinopathy (ICDR) severity scales have laid a solid foundation for research and clinical practice, there is a pressing need for an updated framework that incorporates the latest technological developments into retinal imaging and disease assessment.
